# Effects of cryopreservation and long-term culture on biological characteristics and proteomic profiles of human umbilical cord-derived mesenchymal stem cells

**DOI:** 10.1186/s12014-020-09279-6

**Published:** 2020-05-24

**Authors:** Xufeng Fu, Bo Xu, Jiang Jiang, Xing Du, Xiaoli Yu, Yaping Yan, Shanshan Li, Briauna Marie Inglis, Huiming Ma, Hongyan Wang, Xiuying Pei, Wei Si

**Affiliations:** 1 Key Laboratory of Fertility Preservation and Maintenance of Ministry of Education, Ningxia Medical University, Yinchuan, 750004 China; 2 Yunnan Key Laboratory of Primate Biomedical Research, Institute of Primate Translational Medicine, Kunming University of Science and Technology, Kunming, 650500 China; 3 Department of Obstetrics, The First People’s Hospital of Yunnan Province, Kunming, 650032 China

**Keywords:** Human umbilical cord, Mesenchymal stem cells, Cryopreservation, Long-term culture, Proteomic analysis

## Abstract

**Background:**

Human umbilical cord-derived MSCs (hUC-MSCs) have been identified as promising seeding cells in tissue engineering and clinical applications of regenerative medicine due to their advantages of simple acquisition procedure and the capability to come from a young tissue donor over the other MSCs sources. In clinical applications, large scale production is required and optimal cryopreservation and culture conditions are essential to autologous and allogeneic transplantation in the future. However, the influence of cryopreserved post-thaw and long-term culture on hUC-MSCs remains unknown, especially in terms of specific protein expression. Therefore, biological characteristics and proteomic profiles of hUC-MSCs after cryopreserving and long-term culturing were investigated.

**Methods:**

Firstly, hUC-MSCs were isolated from human umbilical cord tissues and identified through morphology, surface markers and tri-lineage differentiation potential at passage 3, and then the biological characteristics and proteomic profiles were detected and compared after cryopreserving and long-term culturing at passage 4 and continuously cultured to passage 10 with detection occurring here as well. The proteomic profiles were tested by using the isobaric tags for relative and absolute quantification (iTRAQ) labeling technique and differential protein were confirmed by mass spectrometry.

**Results:**

The results showed no significant differences in phenotypes including morphology, surface marker and tri-lineage differentiation potential but have obvious changes in translation level, which is involved in metabolism, cell cycle and other pathways.

**Conclusion:**

This suggests that protein expression may be used as an indicator of hUC-MSCs security testing before applying in clinical settings, and it is also expected to provide the foundation or standardization guide of hUC-MSCs applications in regenerative medicine.

## Background

Mesenchymal stem cells (MSCs) have been regarded as one of the most promising adult stem cells for clinical applications in cell therapy and regenerative medicine due to the capabilities of self-renewal, immunomodulation, multi-lineage differentiation and paracrine function [1]. Moreover, insignificant ethical issues cause MSCs to be seen as more advantageous in clinical applications compared to embryonic stem cells [2]. Since the discovery of MSCs in bone marrow in 1966, various tissues have been reported as the sources of MSCs [3]. The isolation of MSCs from human umbilical cord (hUC) has been recognized as a major alternative source. Normally, postnatal tissues after childbirth are discarded as medical waste, and the harvest and utilization of human umbilical cords is noninvasive and causes negligible bioethics concerns [4]. The hUC-MSCs originate from newborns, while the range of bone marrow derived MSC (BM-MSC) donors’ ages is wide and the harvest process of bone marrow is invasive [5]. A positive correlation between donor ages and the accumulation of mutations in MSCs has been observed in previous studies [6–8]. Moreover, hUC-MSCs show lower immunogenicity after cell transplantation compared to other sources derived MSCs [9]. Therefore, hUC-MSCs show better superiority than BM-MSCs in terms of source and their unique characteristics make hUC-MSCs an extremely valuable candidate for cell therapeutic medicine [5].

Conventionally, the dosages for MSCs transplantations is 10^6^ cells/kg body weight and the total amount of MSCs for one patient is about 10^8^ per cell therapy in clinical trials [10]. Usually, the number of MSCs derived from either autologous or allogeneic tissues is limited, and it is necessary to expand MSCs in vitro before therapy. However, the long-term cultivation of MSCs can result in differentiation-related gene expression and mitochondrial morphology change, reactive oxygen species (ROS) generation and cell senescence, which may deteriorate MSCs features [11]. Therefore, the development of an ideal technique is essential to large-scale MSCs production and storage and it also requires minimal impact on MSCs.

Cell cryopreservation is a widely used technology for long-term storage of cells by cooling the cells to cryogenic temperatures (− 196 °C in liquid nitrogen, for example) [12]. In our previous study, we found that BM-MSCs of a nonhuman primate vitrified with a high level (5.6 M) of the penetrating cryoprotectant either DMSO or ethylene glycol (EG) resulted in changes of a large number of transcripts [13]. Currently, the most widely used method for MSCs cryopreservation is the slow-freezing approach with using a low level of DMSO (1.5 M) as the penetrating cryoprotectant. However, the effects of slow-freezing with a low level of DMSO on the global gene transcripts and proteomics profiles of MSCs have not been studied (Additional file 1: Table S1).

In the present study, we aimed to comprehend the effects of conventional slow-freezing cryopreservation and long-term cultivation on the proteomic profiles of hUC-MSCs. The study will provide a basis for the influence of cryopreservation and cultivation on protein expression, and help facilitate the applications of hUC-MSCs in cell therapeutic medicine.

## Materials and methods

### Ethics statement

The ethical approval was obtained in advance by the Ethics Review Board of Ningxia Medical University and General Hospital of Ningxia Medical University, and informed patient consent for participation was obtained from all subjects.

### Isolation and culture of hUCs derived MSCs

Three hUCs collected from full-term births were used and evaluated separately for this study. The hUC tissues were sanitized with 75% alcohol for 5 min and transferred to the lab within 1 h in Hanks balanced salt solution (HBSS, Sangon biotech, Shanghai, China). The hUCs were cut into 0.5 × 0.5 cm pieces with sterile forceps and curved scissors. The pieces were cultured in sterile 10 mm plastic Petri dishes containing 10 ml of low glucose Dulbecco’s modified Eagle’s medium (DMEM, Gibco BRL, Grand Island, NY, USA) supplemented with 10% (*v*/*v*) fetal bovine serum (FBS, Gibco) and 1% (*v*/*v*) penicillin/streptomycin (Gibco) at 37 °C in an incubator with a humidified atmosphere of 5% CO_2_ and the medium was refreshed every 48 h. A large amount of fibroblast-like cells around the hUCs tissue pieces appeared 1 week later. The remained hUCs tissues were removed and these primary fibroblast-like cells (passage 0) were passaged at 80% confluency by using 0.25% trypsin (Gibco). The cells were resuspended in culture medium at a dilution ratio of 1:3 and expanded on a new plastic Petri dish to passage 1 [14]. The morphology, surface markers and differentiation potency of MSCs were identified at passage 3.

### Morphological and immunophenotypic characterization of hUC-MSCs

The morphological characteristics of hUC-MSCs were assessed under a light microscope (Nikon DIAPHOT 300, Japan) at primary culture and upon passaging in all the experimental groups. The morphological images in this present study were taken at 20 × magnification. The expression of cell surface markers were evaluated using a Human MSC Analysis Kit (BD Biosciences, San Jose, CA) with a C6 flow cytometer (BD Biosciences, San Jose, CA) at 3rd, 4th and 10th passages. Briefly, hUC-MSCs were collected and washed with 500 μL of PBS (containing 3% FBS, PBSF) and the concentration was adjusted at 1 × 10^6^ cells/mL by using a hemacytometer. Then a total of 100 μL of the cell suspension (approximately 5 × 10^5^ cells) was distributed in a 1.5 mL centrifugal tube and incubated with 5 μL (10 μg/μL) of human monoclonal antibodies against a positive (CD44, CD73, CD90 and CD105) and negative cocktail (including CD34, CD45, CD14, CD19, and HLA-DR) at room temperature for 30 min according to the manufacturer’s instructions. Unbound antibodies were washed off with PBS and subsequently the cells were resuspended in 500 μL of PBSF mixture before flow cytometric testing [13].

### Evaluation of the differentiation potential of hUC-MSCs

For adipogenic differentiation, hUC-MSCs were seeded into 24-well plates and cultured for 12 h at a density of 8 × 10^4^ cells per well. Subsequently, the medium was substituted with the adipogenic differentiation medium (Biological Industries, Israel) for 21 days, and the medium was refreshed every 3 days. The induced cells were stained with Oil Red O in a MSCs Adipo-Staining Kit (XP Biomed Ltd., Shanghai, China) according the instructions.

For osteogenic differentiation, hUC-MSCs were seeded into 24-well plates and cultured for 12 h at a density of 4 × 10^4^ cells per well. Subsequently, the medium was substituted with the osteogenic differentiation medium (Biological Industries, Israel) for 21 days, and the medium was refreshed every 3 days. The induced cells were stained with alizarin red solution in a MSCs Osteo-Staining Kit (XP Biomed Ltd., Shanghai, China) according the instructions.

For chondrogenic differentiation, 2 × 10^5^ hUC-MSCs were pelleted in 15-mL centrifuge tubes and cultured with the chondrogenic differentiation medium (Biological Industries, Israel) for 21 days and the medium was refreshed every 3 days. The chondroid pellets were sectioned by a freezing microtome and the slices were stained with toluidine blue in a MSCs Chondro-Staining Kit (XP Biomed Ltd., Shanghai, China) according the instructions [15].

All differentiation evaluations were repeated 3 times.

### Cryopreservation of hUC-MSCs

**Fig. 1:**
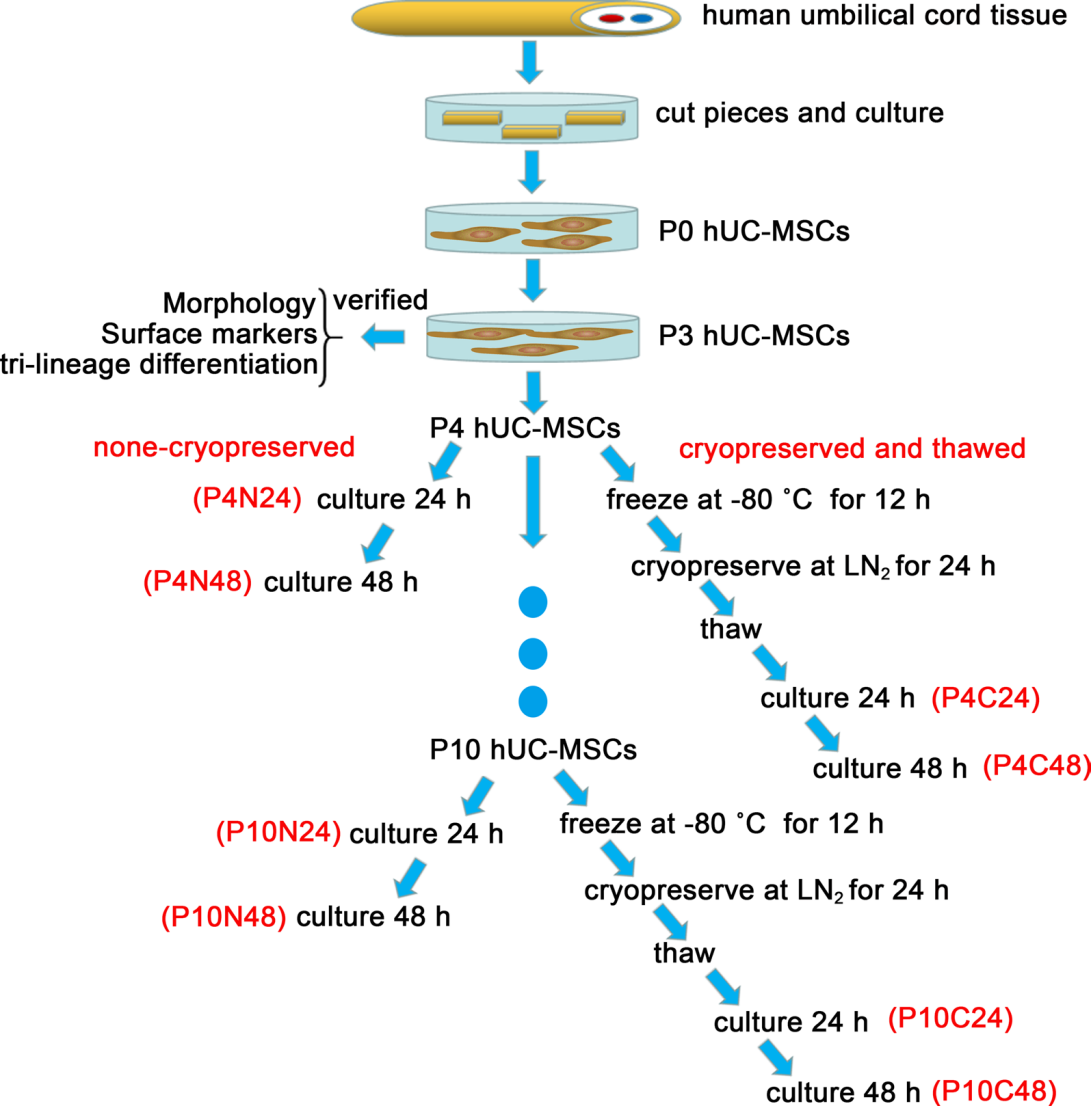
A schematic illustration of the procedure for hUC-MSCs cryopreservation and long-term culture. LN_2_: liquid nitrogen. P4N24: non-cryopreserved and sub-cultured for 24 h at P4. P4C24: cryopreserved and sub-cultured for 24 h at P4, P4N48: non-cryopreserved and sub-cultured for 48 h at P4, P4C48: cryopreserved and sub-cultured for 48 h at P4, P10N24: non-cryopreserved and sub-cultured for 24 h at P10, P10C24: cryopreserved and sub-cultured for 24 h at P10, P10N48: non-cryopreserved and sub-cultured for 48 h at P10, P10C48: cryopreserved and sub-cultured for 48 h at P10

The hUC-MSCs from the three donors were harvested at passage 4 and 10 for the cryopreservation assay when the cells reached 80% confluency. The cell suspension was divided into two equal aliquots at a density of 2 × 10^6^ cells/mL. One of the aliquots without cryopreservation was sub-cultured in fresh medium for 24 h, and cell viability, immunophenotype surface markers, proliferation and metabolic activity were subsequently examined as a non-frozen control. The other cells were cryopreserved by the conventional cell freezing method with the freezing medium composed of DMEM medium supplemented with 10% FBS and 10% DMSO. The mixture of freezing medium and hUC-MSC suspension (1 mL) in a 1.8 mL cryovial containing a density of 1 × 10^6^ cells/mL was cooled at approximately 1 °C/min from 25 to − 80 °C in a freezing container (Nalgene, Rochester, NY) for 12 h and then the cryovials were plunged directly into liquid nitrogen for storage. This is the most commonly used method and equipment for MSCs cryopreservation in laboratories all over the world [16, 17]. After being stored in liquid nitrogen for 24 h, the cells were rapidly warmed by immersing the cryovial in a 37 °C water bath for 5 min. Post-thawed cells were cultured for 24 h for recovery and subsequently evaluated as described in the following assays. The cryopreserved MSCs (abbreviated as “C” from now on) were subcultured for 24 and 48 h at P4 and P10, respectively, and non-cryopreserved MSCs (abbreviated as “N” from now on) cultured for 24 and 48 h at same passages were used as controls. The schematic illustration of the procedure was shown in Fig. 1. A total of 8 groups of hUC-MSCs were involved in this study as follows: non-cryopreserved and sub-cultured for 24 h at P4 (P4N24), cryopreserved and sub-cultured for 24 h at P4 (P4C24), non-cryopreserved and sub-cultured for 48 h at P4 (P4N48), cryopreserved and sub-cultured for 48 h at P4 (P4C48), non-cryopreserved and sub-cultured for 24 h at P10 (P10N24), cryopreserved and sub-cultured for 24 h at P10 (P10C24), non-cryopreserved and sub-cultured for 48 h at P10 (P10N48) and cryopreserved and sub-cultured for 48 h at P10 (P10C48).

### Measurement of cell viability

The viability of cells from P4N24, P4C24, P4N48, P4C48, P10N24, P10C24, P10N48 and P10C48 groups were measured by trypan blue dye (Solarbio, Beijing, China) exclusion assay. Ten μL of cell suspension was mixed 10 μL 0.4% *w*/*v* trypan blue solution for 5 min, and the dead cells were stained and counted with a haemocytometer under a light microscope.

### Proteomics analysis and targeted quantitative detection of hUC-MSCs

The cells from non-cryopreserved groups (P4N24, P4N48, P10N24, P10C24, and P10N48) and cryopreserved groups (P4C24, P4C48, P10C24 and P10C48) groups were collected for proteomic profile detection. The proteomics procedures were performed by PTM Biolabs Lnc. (Hangzhou, Zhejiang, China). Briefly, a cell sample was sonicated by high intensity ultrasonic processor in lysis buffer of urea and protease inhibitor cocktail, and the remaining cell debris was removed by centrifugation. The protein concentration of the supernatant was collected and quantified with BCA kit (Thermo Fisher, USA), and prokaryotic standard protein was added for detecting quality control [18]. Then, the protein solution was reduced with dithiothreitol and alkylated with iodoacetamide, and the urea concentration was diluted by adding tetraethylammonium bromide, and then the protein samples were digested by trypsin. After trypsin digestion, the peptide was desalted and processed according to the manufacturer’s protocol for TMT/iTRAQ kit. The tryptic peptides were fractionated into fractions by high pH reverse-phase HPLC using Agilent 300 Extend C18 column, and the peptides were dissolved by acetonitrile and analyzed by tandem mass spectrometry in Q ExactiveTM Plus (Thermo) coupled online to the EASY-nLC 1000UPLC. The data of tandem mass spectrometry were processed using Maxquant search engine (v.1.5.2.8) and annotation results from database were collected for analysis. Quantitative analysis of differentially expressed proteins was also performed depending on Parallel Reaction Monitoring (PRM) technology by PTM Biolabs Lnc. according to their commercial manufacturer’s instructions. The pre-processing of samples as well as proteomics analysis, besides, quantitative analysis was used as a standard to quantify special protein from samples.

### Statistical analysis

The data from viability and markers expression were significantly analyzed statistically using Graphpad software (GraphPad Prism; Graphpad Software, Inc., San Diego, CA) and presented as the mean ± SD. Comparative assessment of mean value among various factors was performed using ANOVA and unpaired *t* test and a *P*-value < 0.05 was considered statistically significant.

Differential protein screening was based on a 1.3-fold change, and the ratio between the samples at more than 1.3-fold change or less than 1/1.3-fold change were considered up-regulated or down-regulated trend *P*-value < 0.05. For further study of the hierarchical clustering, all the categories were obtained and enriched in clusters depending on *P*-value < 0.05, and the cluster membership were visualized by a heat map using the “heatmap.2” function from the “gplots” R-package. Proteins were classified by Gene Ontology (GO) annotation, which was derived from the UniProt-GOA database (www. http://www.ebi.ac.uk/GOA/). The pathways of different proteins were classified according to the Kyoto Encyclopedia of Genes and Genomes (KEGG) database website.

Identified proteins domain functional description was annotated by InterProScan based on InterPro (http://www.ebi.ac.uk/interpro/) domain database. These enrichment analyses were tested according to the database of identified proteins and employed two-tailed Fisher’s exact test, all terms with corrected *P*-values < 0.05 were considered significantly enriched differentially expressed proteins.

## Results

### Basic characterization of hUC-MSCs

**Fig. 2:**
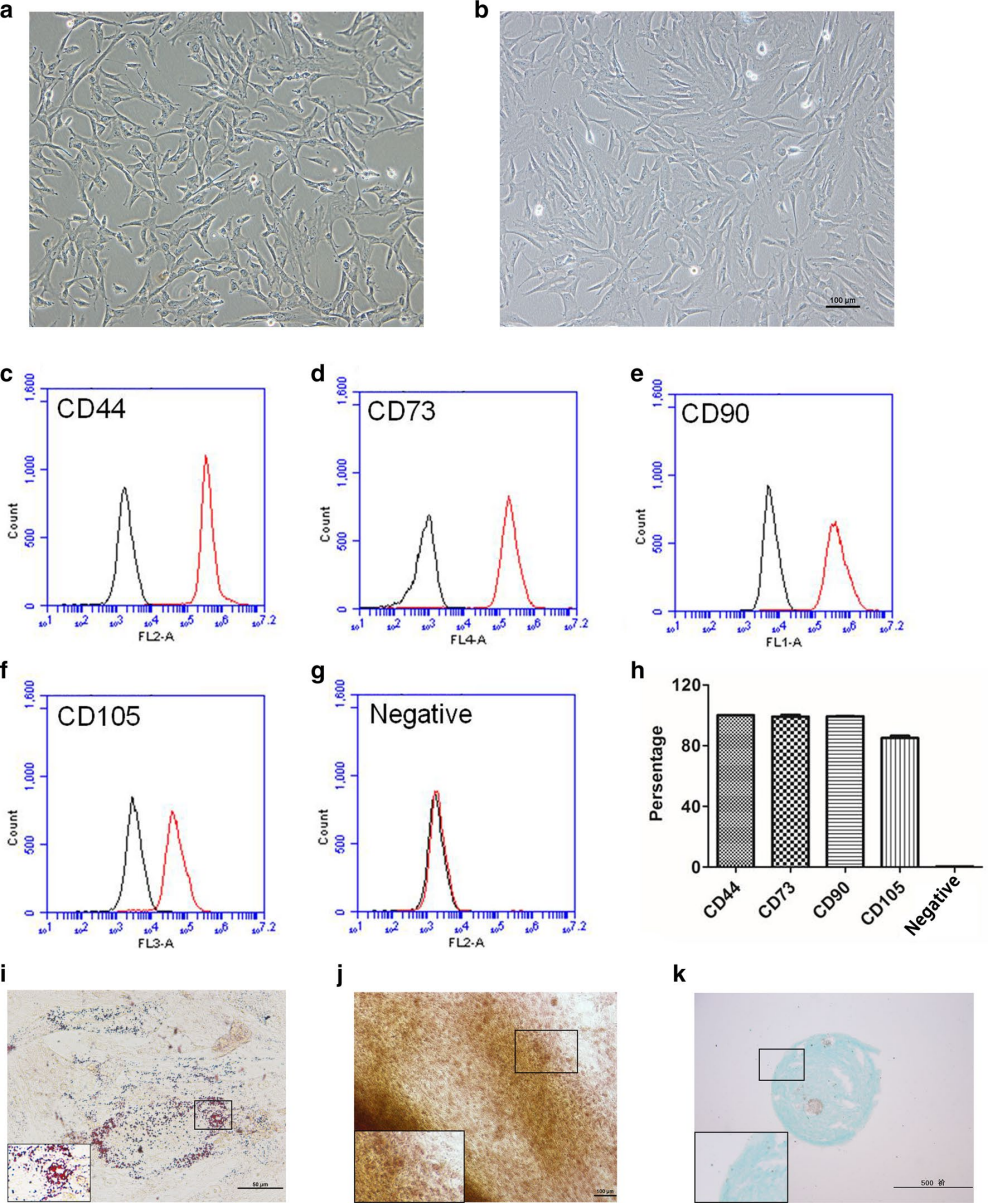
Fibroblast-like morphology of MSCs at passage 0 (**a**) and passage 3 (**b**). Scale bars: 100 μm. **c**–**g** Surface markers expression on human umbilical cord-derived MSCs at passage 3 analyzed using flow cytometry. Black lines represent isotype control. **h** Quantitative profile of surface markers expression (n = 3). **i**–**k** Differentiation potency of MSCs at passage 3. **i** Adipogenic differentiation (oil red staining, × 200); **j** Osteogenic differentiation (alizarin red staining, × 100); **k** Chondrogenic differentiation (alcian blue staining, × 50). Scale bars: **i** was 50 μm, **j** was 100 μm and **k** was 500 μm

During primary culture, the spindle-shaped and fibroblast-like cells were dispersed around the shredded umbilical cord tissues. These cells grew adhesively in plastic dishes in a scattered manner, formed colonies and appeared heterogeneously regarded as hUC-MSCs of passage 0 (P0, Fig. 2a). The hUC-MSCs colonies at passage 0 were extended to passage 3 (P3) with subsequent subculture, and the P3 hUC-MSCs also showed a spindle-shaped and fibroblast-like morphology (Fig. 2b). The surface marker profiles of the hUC-MSCs were analyzed at P3 by flow cytometry. The percentage of positively expressed surface markers was 100.0 ± 0.0% of CD44, 99.3 ± 0.2% of CD73 and 85.0 ± 1.4% of CD105, and the percentage of negative expressed cocktail surface markers was 0.2 ± 0.1% (Fig. 2c–h). After adipogenic, osteogenic and chondrogenic differentiation, the P3 hUC-MSCs formed numerous neutral lipid droplets in the cytoplasm identified by Oil Red O staining (Fig. 2i), mineral accumulation and bone nodules formation was identified by alizarin red staining (Fig. 2j) and proteoglycan and hyaluronic acid accumulation was identified by alcian blue staining (Fig. 2k).

### Effect of long-term culture and cryopreservation on the biological characteristics of hUC-MSCs

**Fig. 3:**
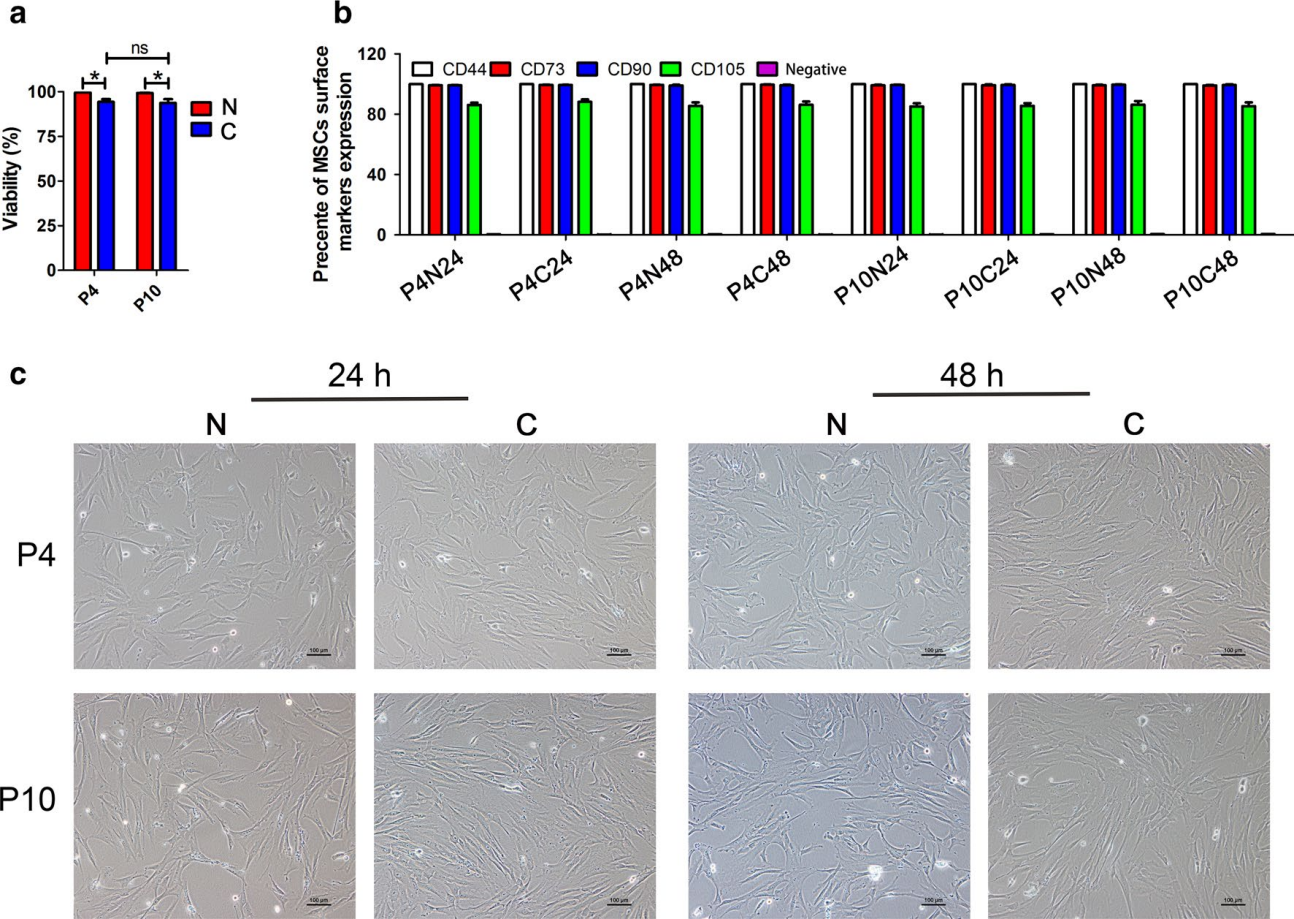
Comparison of the cell viability (n = 3) (**a**), surface markers expression (n = 3) (**b**) and morphology (**c**) between non-cryopreserved (N) and cryopreserved (C) groups after being sub-cultured for 24 h or 48 h at P4 and P10, respectively

**Fig. 4:**
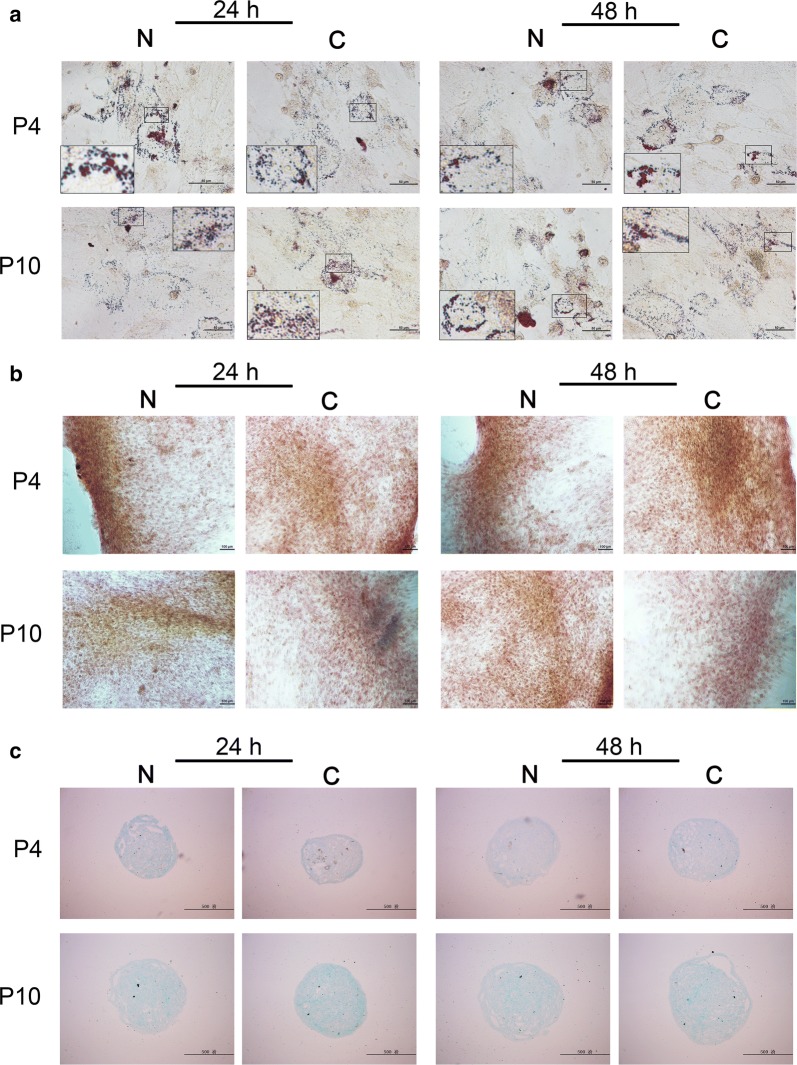
Comparison of adipogenic (**a**), osteogenic (**b**) and chondrogenic (**c**) differentiation potency between non-cryopreserved (N) control and cryopreserved (C) groups after being sub-cultured for 24 h or 48 h at P4 and P10, respectively

As shown in Fig. 3a, the viability of hUC-MSCs were significantly decreased after instant freezing and thawing (abbreviated as “C” groups from now on) compared to non-cryopreserved control (abbreviated as “N” groups from now on) either at passage 4 (P4, N vs. C, 99.61 ± 0.22% vs. 94.42 ± 1.53%) or passage 10 (P10, N vs. C, 99.44 ± 0.51 vs. 93.82 ± 2.13%). After a sub-culture for 24 h or 48 h post thawing, the hUC-MSCs either at P4 or P10 remained to possess a high level expression of positive surface markers (CD44, CD73, CD90 and CD105) and barely expressed negative markers of MSCs, and no significant differences were observed compared to non-cryopreserved controls. The results suggested that the expression of surface markers was not affected by cryopreservation and long-term culture (Fig. 3b). The morphology of cells from non-frozen control and cryopreserved groups following a 24 h and 48 h sub-culture post thawing are shown in Fig. 3c. No obvious morphological changes were observed among the eight groups. Similar to the cells from control groups, the differentiation potency of hUCs from N24, N48, C24 and C48 groups at P4 and P10 showed no obvious difference evaluated by adipogenic (Fig. 4a), osteogenic (Fig. 4b) and chondrogenic differentiation (Fig. 4c).

### Effect of cryopreservation and long-term culture on proteome profiles of hUC-MSCs

**Fig. 5:**
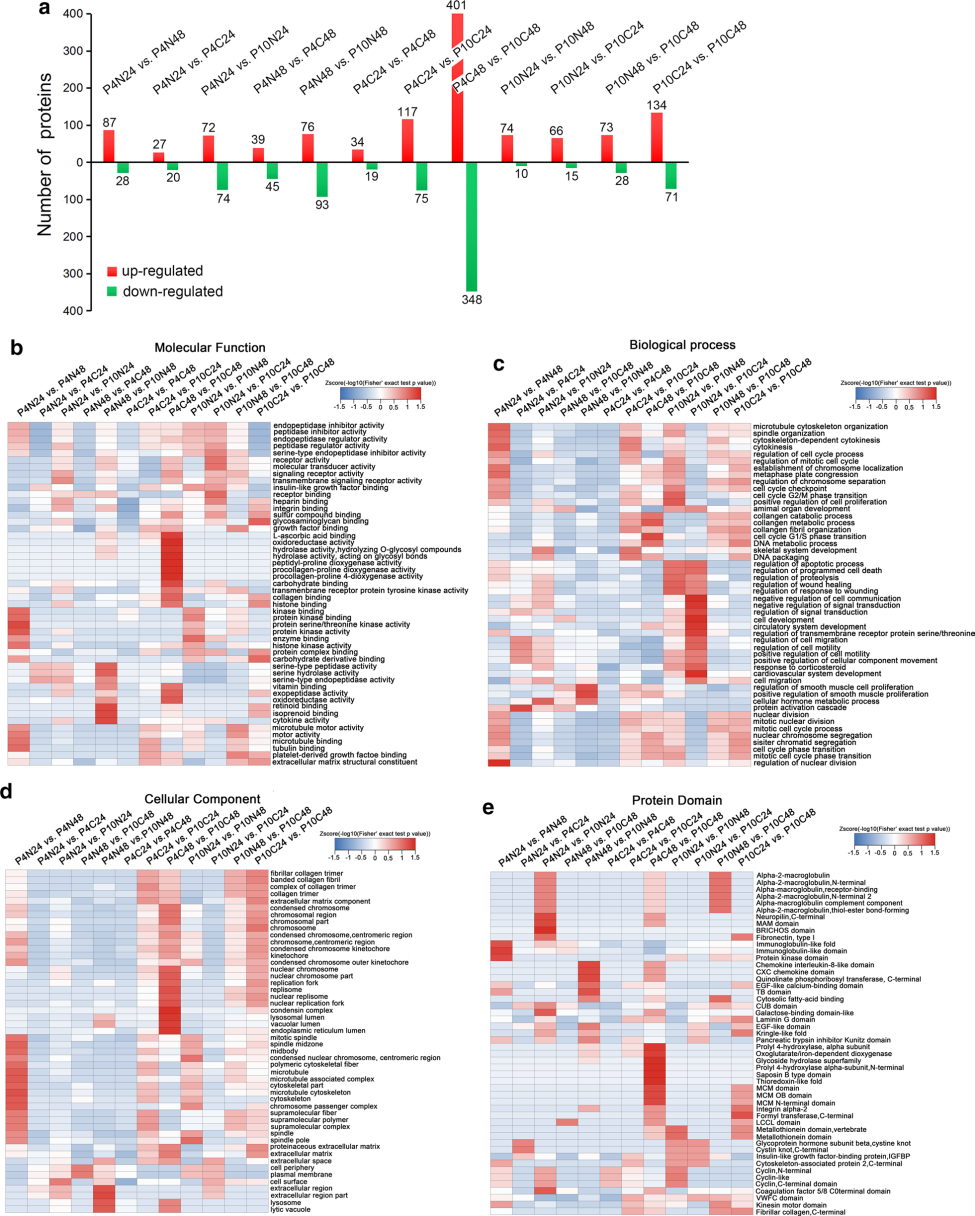
The number and the GO heatmaps of differentially expressed proteins. **a** The histogram of differentially expressed proteins. The heatmap of differential proteins enriched pathways in molecular function (**b**), biological process (**c**), cellular component (**d**) and protein domain (**e**)

The number of significantly modulated proteins of hUC-MSCs among the 8 groups are summarized in Fig. 5a. These results indicated that the proteome profiles of hUC-MSCs were affected by either long-term culture or cryopreservation. The functional enrichment analysis according to Gene Ontology (GO) of differentially expressed proteins among hUC-MSCs from the 8 groups was summarized in Fig. 5b–d. The heatmap graphs of the GO display the distribution of the biological terms presented in molecular function (Fig. 5b), biological process (Fig. 5c) and cellular component (Fig. 5d). In molecular function, protein kinase activity and microtubule motor activity were affected by continuous culture from 24 h to 48 h at P4 without cryopreservation (P4N24 vs. P4N48). Retinoid, isoprenoid binding and cytokine activity were affected by long-term culture from P4 to P10 without cryopreservation (P4N48 vs. P10N48). Oxidoreductase, hydrolase and peptidyl-proline dioxygenase were affected by cryopreservation and sub-culture for 48 h at P10 compared to P4 (P4C48 vs. P10C48), which were shown in Fig. 5b. In biological process as shown in Fig. 5c, regulation of nuclear division was affected by continuous culturing from 24 h to 48 h at P4 without cryopreservation (P4N24 vs. P4N48), protein activation cascade was affected by cryopreservation and sub-culture for 24 h at P4 compared to non-cryopreserved and sub-cultured for 24 h (P4N24 vs. P4C24), regulation of smooth muscle cell proliferation was affected by long-term culture from P4 to P10 without cryopreservation (P4N48 vs. P10N48), cell proliferation and programmed cell death were affected by continuous culturing from 24 h to 48 h at P10 (P10N24 vs. P10N48), and cell communication and signal transduction were affected by post-thawing and sub-culturing for 24 h at P4 compared to non-cryopreservation at P10 (P10N24 vs. P10C24). In cellular component as shown in Fig. 5d, cytoskeleton and chromosome passenger complex were affected by continuous culture from 24 h to 48 h at P4 (P4N24 vs. P4N48), extracellular region and lysosome were affected by long-term culture from P4 to P10 without cryopreservation (P4N48 vs. P10N48), nuclear replication fork, lysosomal and endoplasmic reticulum lumen were affected by cryopreservation and sub-culture for 48 h at P10 compared to P4 (P4C48 vs. P10C48).

In order to further analyze the effect of cryopreservation on hUC-MSCs function, differential proteins enriched in biological processes of GO classification having known identities in MSCs functions. The functions of these differentially hUC-MSCs proteins are listed in Table 1, which are associated with differentiation, immunoregulation, wound healing and regeneration, apoptotic signaling pathway, oxidation resistance, cartilage development, regulation of cytokine production, cell migration and others. Specific protein information and the fold of change in different groups were shown in Table 2.

**Table 1:** Biological processes classification of differential identified proteins in MSCs

Biological processes	Gene name
Differentiation	GATA6, DKK1, STC1, PDGFRB, COL5A2, FST, CCNB1, AURKA, TOP2A, INHBA, COL1A1, ANLN, JUN, SEMA7A, NCAM1, COL12A1, NRP2, FBN2, HGF
Immune system regulation process	TNFAIP3, KIF2C, PTX3, TMBIM1, IGHG1, JUN, NDRG1, NCAM1, MYO10, KIF22, COL1A1, RACGAP1, SEMA7A, KIF11, INHBA, MT2A, FST, C3, GEM, TOP2A, SERPINE1, KIF23, ANLN, PDCD1LG2, CRISPLD2, JUN
Wound healing and regeneration	TNFAIP3, SERPINB2, GATA6, MKI67, SERPINE1, F3, FOSL1, AURKA, COL1A1, DCN, NRP2, HGF, JUN, C3, PDGFRB, CCNB1, TFPI2, HIST2H3A, CCNA2
Apoptotic signaling pathway	BIRC5, STK17B, F3, TNFAIP3, SERPINE1, INHBA, TMBIM1, TIMP3, TOP2A, CHEK1, HGF, PDGFRB, TPX2, GATA6, SERPINB2, AURKA, CCNB1, TNFAIP3, NUAK1, FOSL1, AURKB, CPEB4, PLK1, JUN, ARAF, AMIGO2
Myeloid cell differentiation and ossification	INHBA, FBN2, STC1, COL5A2, JUN, HGF, COL1A1, SEMA7A
Oxidation resistance	TNFAIP3, PTX3, NDRG1, TIMP3, SERPINE1, COL1A1, NDRG1, STK17B,AURKA, PLK1, PDGFRB, CPEB4, AURKB, AMIGO2, TMBIM1, JUN, STC1, ARAF, GATA6, HGF, FOSL1, CCNB1, TOP2A, F3, CPEB4, BIRC5, SERPINB2, INHBA, CCNA2
Adaptive immune response	TNFAIP3, IGHG1, MYO10, DCN, C3, HGF, PDCD1LG2, JUN, SEMA7A, FST, INHBA, PTX3, TOP2A, SERPINE1, NCAM1, ANLN, MT2A
Inflammatory response	TNFAIP3, PTX3, SERPINE1, PDCD1LG2, SEMA7A, F3, HGF, C3
Interferon-gamma-mediated signaling pathway	PDCD1LG2, NCAM1, INHBA, MT2A
Cartilage development	STC1, BNC2, LUM, COL1A1, STC1, COL1A1, MEX3C
Regulation of cytokine production	GATA6, LUM, TNFAIP3, SERPINE1, PDCD1LG2, SEMA7A, INHBA, HGF, C3
Angiogenesis	SERPINE1, GATA6, PDGFRB, F3, JUN, HGF, NRP2, C3
Antigen processing and presentation	RACGAP1, TNFAIP3, CCNA2, KIF22, INHBA, KIF11, KIF2C, KIF23
Cell migration	F3, STC1, SERPINE1, SMURF2, DCN, COL1A1, PDGFRB, HGF, NRP2, JUN
Transforming growth factor beta1 production	GATA6, COL1A1, C3, LUM, JUN, SERPINE1, TNFAIP3, PDCD1LG2, SEMA7A, HGF, INHBA
Response to growth factor	PDGFRB, GATA6, NRP2, DKK1, SHCBP1, SHCBP1, CCNA2, SMURF2, COL1A1, SMURF2, JUN, FBN2, HGF, DCN, LUM
Aging	CHEK1, SERPINE1, AURKB, PDGFRB, DCN, JUN
Regulation of endothelial cell proliferation	SERPINE1, GATA6, DCN, TNFAIP3, THBS2, HGF, F3, C3, JUN, NRP2

**Table 2:**
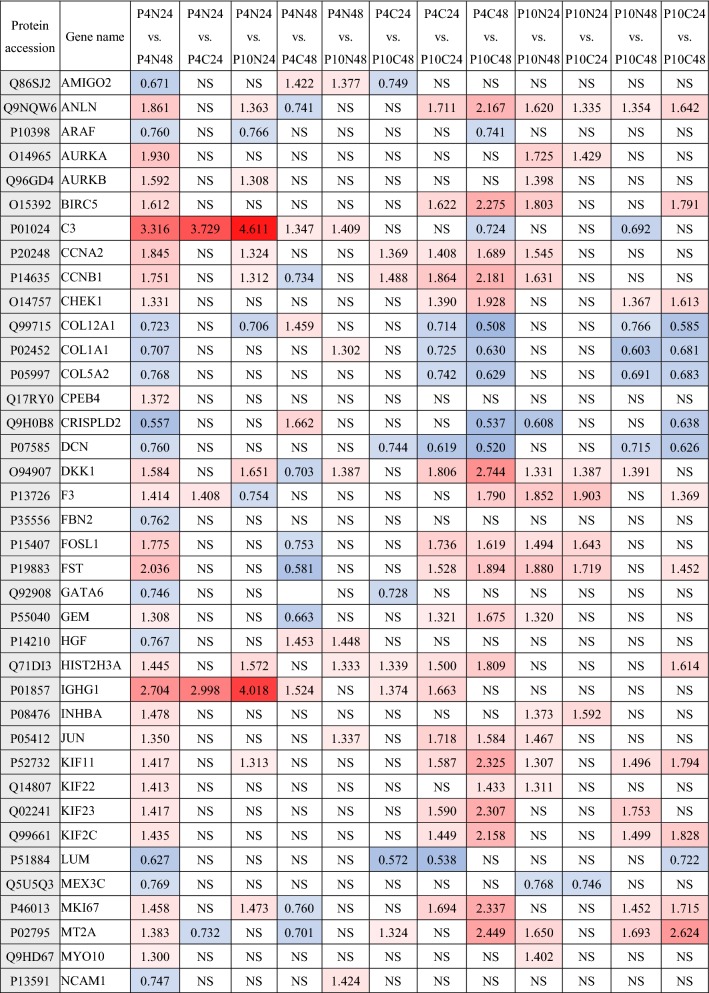

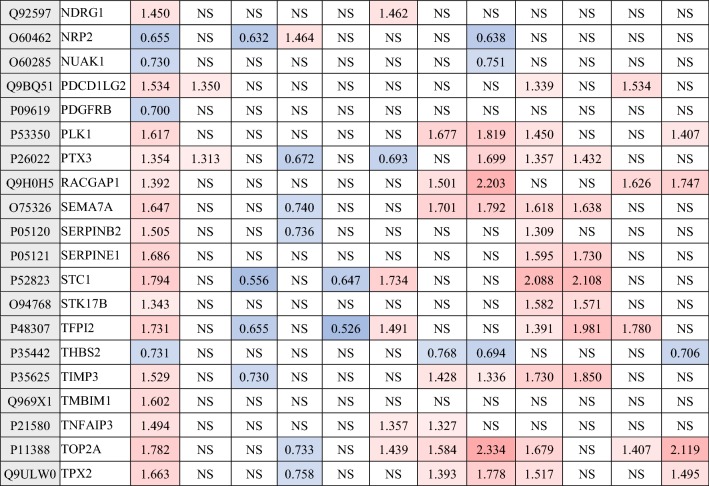
Differential proteins associated with MSCs function

Red is up-regulation and blue is down-regulation, NS is no significant difference

Protein domain was analyzed after cryopreservation and sub-culture for 24 h and 48 h at P4 and P10, respectively, compared with non-cryopreserved groups as shown in Fig. 5e. The results showed that immunoglobulin-like fold domain was affected by the continuous culture from 24 h to 48 h at P4 without cryopreservation (P4N24 vs. P4N48). BRICHOS domain and galactose-binding domain-like were affected by long-term culture from P4 to P10 without cryopreservation and sub-culture for 24 h (P4N24 vs. P10N24). Chemokine domain was affected by continuous culturing from by long-term culture from P4 to P10 without cryopreservation and sub-culture for 48 h (P4N48 vs. P10N48). Hydroxylase, iron-dependent dioxygenase, glycoside hydrolase superfamily and thioredoxin-like fold domain were affected by cryopreservation and sub-culturing 48 h at P10 compared to P4 (P4C48 vs. P10C48).

**Fig. 6:**
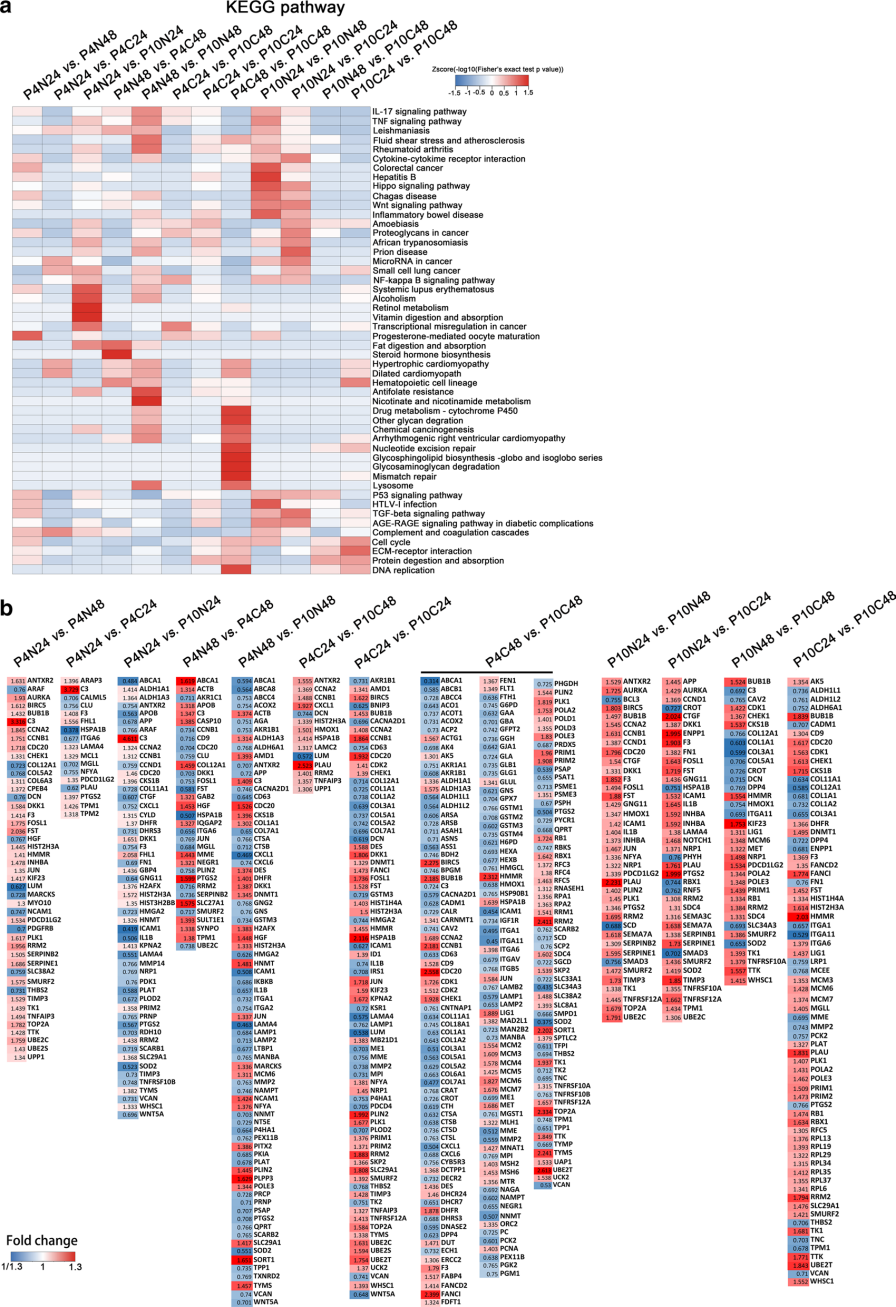
Heatmap of pathways (**a**) and differentially expressed proteins (**b**) enriched according to KEGG database among the eight groups

In addition, differentially expressed proteins were also analyzed by KEGG (Kyoto Encyclopedia of Genes and Genomes) to show the network of pathway interactions (The raw data of differentially expressed proteins enriched in KEGG database as shown in Additional file 1: Table S1). The results as shown in Fig. 6a indicated that progesterone mediated oocyte maturation, complement and coagulation cascades and protein digestion related pathways were affected by continuous culturing from 24 h to 48 h at P4 (P4N24 vs. P4N48) without cryopreservation. Retinol metabolism and vitamin absorption related pathways were affected by long-term culture from P4 to P10 without cryopreservation and sub-culture for 24 h (P4N24 vs. P10N24). Steroid hormone biosynthesis related pathways was effected by post-thawing and sub-culturing for 48 h compared to non-cryopreservation and sub-culture for 48 h at P4 (P4N48 vs. P4C48). Nicotinamide metabolism, antifolate resistance and staphylococcus aureus infection related pathways (Not contaminated) were affected by long-term culture from P4 to P10 without cryopreservation and sub-culture for 48 h (P4N48 vs. P10N48). Glycosaminoglycan degradation and DNA replication related pathways were affected by cryopreservation and sub-culture for 48 h at P10 compared to P4 (P4C48 vs. P10C48). In addition, the differentially expressed proteins of the enriched KEGG pathway were listed as Fig. 6b, the red and blue present up-regulaed and down-regulated proteins, respectively. These results indicated that the expression of hUC-MSCs proteins which are involved in many pathways were changed by cryopreservation as well as long-term culturing at P4 and P10.

### Verification of cryopreservation and long-term culture induced candidate proteins by PRM

The differentially expressed proteins were separated into several categories according to their functions by GO and KEGG enrichment analysis, to validate the results of MS and to compare the influence mechanisms of cryopreservation and long-term culture on hUC-MSCs, we used PRM analysis to assess the abundance of 14 candidate proteins whole abundance changes in response to hUC-MSCs cryopreservation and long-term culture as determined by TMT. The 14 differentially expressed proteins as well as enriched various pathways were selected from 4 groups (P4N24 vs. P4C24, P4N24 vs. P10N24, P4C24 vs. P10C24, P10N24 vs. P10C24) and involved in tdioxygenase activity, cell development, extracellular matrix, oxidoreductase activity, reproductive process, hydrolase activity, ATP binding, protein kinase activity, immune process, cell growth and division. As shown in Table 3, 14 proteins in PRM analysis was consistent with the results of TMT-based quantitation results. Although the fold changes of SMTN in P4C24/P10C24, SEMA7A in P4C24/P10C24 and P10N24/P10C24 analyzed by PRM more than TMT, whereas the TMT and PRM results all showed a rising trend. Our PRM results were in consistent with the data from TMT analysis (Table 3), which further confirmed the credibility of the proteomics data.

**Table 3:** Comparison of the quantification results between TMT and PRM of the 14 candidate different expression proteins

Protein accession	Proteins	Signature peptides	P4N24/P4C24		P4N24/P10N24		P4C24/P10C24		P10N24/P10C24	
			TMT	PRM	TMT	PRM	TMT	PRM	TMT	PRM
Q8IVL6	P3H3	DLETPPHWAAYDTGLELLGR	1.03	1.17	0.96	1.06	0.91	0.86	0.98	0.96
P53814	SMTN	AQEIEAATLAGRLQDGTPQAALSPLTPAR	1.04	1.04	1.23	1.27	1.92	3.29	1.63	2.68
Q96CG8	CTHRC1	QCSWSSLNYGIDLGKVLFSGSLR	1.47	2.08	0.89	0.81	0.61	0.39	1.01	1.00
O75326	SEMA7A	DPYCGWDQGR	1.06	1.09	1.10	1.08	1.70	3.03	1.64	3.08
P35354	PTGS2	SHLIDSPPTYNADYGYKSGLDDINPTVLLK	1.40	2.79	0.57	0.50	0.81	0.53	2.00	2.99
Q9NQW6	ANLN	LLLIATGKGFLTIFEDVSGFGAWHR	1.06	1.08	1.36	1.32	1.71	2.44	1.34	1.99
P58335	ANTXR2	VSPVGETYIHEGLKLDALWALLR	0.87	0.97	0.75	0.70	1.18	1.17	1.36	1.63
P48307	TFPI2	LQVSVDDQCEGSTEKTCDAFTYTGCGGNDNNFVSR	1.08	0.96	0.66	0.47	1.20	1.23	1.98	2.51
Q13642	FHL1	FWHDTCFR	1.56	1.45	2.06	2.00	1.69	2.53	1.28	1.84
Q02241	KIF23	ALLQEFDNAVLSK	0.96	1.05	1.29	1.49	1.59	1.82	1.18	1.28
Q9H0H5	RACGAP1	SIGSAVDQGNESIVAK	0.97	0.87	1.25	1.56	1.50	2.47	1.17	1.38
P53350	PLK1	LILYNDGDSLQYIER	0.96	1.01	1.27	1.89	1.68	2.70	1.27	1.44
P00749	PLAU	FEVENLILHK	0.62	0.42	0.43	0.27	1.23	1.50	1.76	2.30
O00762	UBE2C	GISAFPESDNLFKLSLEFPSGYPYNAPTVK	1.02	1.05	1.27	1.52	1.63	2.06	1.31	1.42

## Discussion

Human umbilical cord-derived MSCs are promising seeding cells in cell therapy and regenerative medicine due to their unique advantages. Cryopreservation plays an important role in the maintenance of MSCs function and avoids adverse effects caused by long-term culture [19]. DMSO is a widely used penetrating cryoprotectant for MSCs cryopreservation when using the conventional slow freezing protocol. Although efforts for the reduction of DMSO concentrations have been made to alleviate the adverse reactions of DMSO and decreased DMSO concentration (as low as 2% combined with other cryoprotectants) has been successfully employed [20], the viability of MSCs cannot be guaranteed. In addition, the combination of multiple penetrating cryoprotectants is not conducive to understand the adverse mechanisms of each cryoprotectant on cell recovery or engraftment. In our previous study, DMSO and ethylene glycol (EG) have been used for vitrification of MSCs, and the results showed that the viability of cells vitrified by DMSO is less than those by EG. However, the transcripts of larger numbers of genes affected by EG are much more than those by DMSO [13]. Therefore, the method of conventional slow freezing method by using 10% DMSO was selected in the present study and it is still the most widely used method at present [16, 17]. In regard to the store period (24 h) of MSCs in liquid nitrogen, whether long-term storage more than 24 h will have more profound effects remains need to be further studied [13].

In this present study, the conventional slow freezing method using 10% DMSO was used for MSC cryopreservation. The freezing and thawing process decrease the viability of cells either at P4 (94.42 ± 1.53%) or P10 (93.82 ± 2.13%). In previous studies, Fong et al. reported that hUC-MSCs viability was 85–90% after thawing by using the same slow cooling method [21], and Woods et al. reported the post-thaw viability of human MSCs was about 91% by using 1.0 M (about 7.1%, *w*/*v*) and 1.5 M (about 10.65%, *w*/*v*) DMSO freezing with this method [22]. Our results showed similar viabilities compared to the previous studies. Although the conventional slow freezing method has been widely used and can also obtain better viability, profound influence of the freezing process and cryoprotectant on the transcript and protein function of hUC-MSCs remains unknown.

Conventionally, the morphology, surface marker expression and tri-lineage differentiation potency are regarded as a “gold standard” for identifying MSCs according to the International Society for Cellular Therapy. In this study, there are no significant differences between non-cryopreserved and post-thaw following sub-culture 24 or 48 h in morphology, surface markers and tri-lineage differentiation potency at P4 and P10. Hence, these results concluded that cryopreservation and long-term culture did not affect the characteristics of hUC-MSCs, which are consistent with previous studies [13, 23]. To our knowledge, almost all of the studies have shown that cryopreservation does not affect the morphology, surface markers and differentiation potency as description in a review [24] and proven by our previous [13] and present study. However, our previous study revealed that though the morphology, surface markers and tri-lineage differentiation potency of MSCs were not affected by cryopreservation, the global gene expression was affected either vitrified with DMSO or EG as a cryoprotectant [13]. In the present study, many protein’s expression was affected by cryopreservation and long-term culture revealed by the proteomics analysis. A total of 47 and 81 proteins expressed were affected by freezing and thawing at P4 (P4N24 vs. P4C24) and P10 (P10N24 vs. P10C24), respectively, as well as cell communication and signal transduction were obviously affected though GO analysis. Therefore, in our opinion, the traditional identification standards based on qualitative detection (post thaw viability, morphology, surface markers and tri-lineage differentiation potency) may be insufficient for the evaluation of the change of biological characteristics after cryopreservation or environmental stimulus during long-term culture. Therefore, it is necessary to explore quantitative methods for MSCs quality evaluation such as a protein targeting quantification method in preclinical or clinical application.

Previous studies have reported that cryopreservation can affect the immunomodulatory properties of MSCs, and the levels of heat shock proteins increased and the inflammatory response was impaired within 24 h after thawing. However, these studies considered that the function of MSCs would be completely recovered after 24 h of culturing [25–27]. The protein expression recovery of cryopreserved MSCs is essential to maintain their properties after transplantation in vivo. In this present study, the proteomics profile showed that the 47 and 81 proteins of hUC-MSCs were affected by freeze–thawing and a 24 h sub-culture at P4 and P10, respectively. In this study, two time points (24 and 48 h) were chose in this study because over-time culture can induce over-confluency of hUC-MSCs that is not conducive to evaluate the status of cells, and hUC-MSC passage with fresh culture medium contains serum can affect many proteins expression, which may not reflect the true status of cells after thawing [28]. In P4, the different proteins were enriched in microRNA in cancer, small cell lung cancer, hypertrophic cardiomyopathy and dilated cardiomyopathy due to the proteins such as TIMP3 (Metalloproteinase inhibitor 3), ITGA6 (Integrin alpha-6) and TPMs (Tropomyosins) were affected by culturing from 24 h to 48 h (P4N24 vs. P4N48) and freeze–thawing for culturing 24 h (P4N24 vs. P4C24), and these gene were clustered in pathway of those disease. TIMP3, ITGA6 and TPM are involved in the extracellular matrix, cytoskeleton and cell adhesion that directly related to the cellular regular function, and these genes change may be caused by cryopreservation or cryoprotectant, and cryopreservation could affect surface adhesion molecules had been reported [29]. It is indicated that TIMP3, ITGA6 and TPM may be good markers to detecting impairment of cell function which is still need to be further studied. Many studies have shown that extracellular matrix, cytoskeleton and cell adhesion are connected with lung cancer and cardiomyopathy. TIMP-3 inhibits the activity of metalloproteinases that play important roles in development and progression of lung tumors [30]. TIMP-3 is up-expressed in cardiac fibroblasts and cardiomyocytes but down-expressed in the failing heart [31]. Early studies have reported that ITGA6 is involved in the occurrence and development of lung cancer [32]. It is reported that ITGA6 corresponds to the activation of regeneration involving an epithelial-mesenchymal transition in adult heart [33]. TPM is a potential marker in lung cancer diagnosis [34], and the latest study showed TPM pseudo-phosphorylation results in dilated cardiomyopathy [35]. However, the relationship between cryopreservation of hUC-MSCs after long-term culture and diseases including cancer and cardiomyopathy remains unknown and need to be further studied.

The complement and coagulation cascades were alleviated by sub-culturing from 24 h to 48 h after freeze–thawing compared with the non-cryopreserved group with a sub-culture for 24 h or 48 h parallelly. Meanwhile, the proteins of fat digestion and absorption, steroid hormone biosynthesis, and hematopoietic cell lineage pathways were affected (P4N24 vs. P4C24 and P4N48 vs. P4C48). In P10, many pathways including cytokine–cytokine receptor interaction, hippo signaling pathway, wnt signaling pathway, microRNA in cancer, small cell lung cancer, NF-kappa B signaling pathway and others were significantly alleviated by sub-culturing from 24 h to 48 h after freeze-thawing (P10N24 vs. P10C24 and P10N48 vs. P10C48). These results indicated that the effect of cryopreservation on the protein expression of MSCs at P10 was greater than those at P4. For example, related proteins of complement and coagulation cascades including CLU (Clustering), PLAU(Urokinase-type plasminogen activator), C3 (Complement C3) and F3(Tissue factor) were not recovered until a sub-culture to 48 h at P4, and related proteins of Th17 cell differentiation IL-1B and SMAD3 were not recovered until a sub-culture to 48 h at P10, it maybe that serum or nutritional components for hUC-MSCs growth was less with consumption, and this would cause interference in the expression of a variety of proteins [28, 36]. These results suggest that properly prolonging the time of continuous culture after freeze-thawing can alleviate the effect of cryopreservation on the change of proteins expression. In addition, rare studies have reported that cryopreservation reduces the homing/engraftment potential of MSCs by poor binding to the extracellular matrix such as fibronectin and the immunosuppression ability of MSCs play an important role in MSCs homing/engraftment. However, the knowledge about the recovery status of the main immunoregulation proteins of MSCs after cryopreservation and sub-culture is poor [27, 37].Therefore, it is necessary to sub-culture and recover the functional proteins of hUC-MSCs after cryopreservation and before transplantation, and the optimal recovery methods for MSCs are still need to be further explored.

The proliferation of MSCs is limited during long-term culture and the MSCs exhibit a aberrant phenotype of irregular flattened geometry and enlarged size [38]. Yang et al. found human bone marrow-derived MSCs undergo senescence during extensive passage and result in morphological, phenotypic and genetic changes from P4 to P8 [38]. De Witte et al. reported that long-term expansion induced aging of hUC-MSCs exhibiting stable phenotype but reduced immunosuppressive properties from P4 to P12 [39]. Facchin et al. reported that umbilical cord Wharton’s Jelly-derived MSCs showed higher antioxidant ability to senescence than human adipose tissue-derived MSCs at high subculture passages, and they considered that the age of tissue donors is likely to be the main cause of senescence [40]. Moreover, recently, studies found that transcriptome and epigenetic regulations changes of hUC-MSCs occurred during long-term expansion [41, 42]. These studies not only indicated that long-term culture and expansion induces aging of hUC-MSCs as well as genes expression changed, but also suggested that the antioxidant ability of hUC-MSC is superior to others that were derived from human adult such as bone marrow and adipose tissue. In this present study, the morphology, surface markers expression, tri-lineage differentiation potency and proteomic analysis of hUC-MSCs were evaluated after long-term culturing and expanding from P4 to P10, and the results showed that the morphology, surface markers and differentiation potency were not affected but large scale of proteins were changed from P4 to P10, which involve in proteins related to cell cycle and P53 pathways including CCNB1(G2/mitotic-specific cyclin-B1), CCND1(G1/S-specific cyclin-D1), CHEK1 (Serine/threonine-protein kinase Chk1), RRM2(Ribonucleoside-diphosphate reductase subunit M2), SERPINE1(Plasminogen activator inhibitor 1) and P53 pathway has been reported to relate to aging of MSCs in previous studies [6, 43, 44]. Superoxide dismutase 2 (SOD2) has been reported to participate in the aging of MSCs [45, 46]. In the present study, superoxide dismutase 2 (SOD2) is up-regulated in MSCs at P10 compare to those at P4, which indicated that oxidative stress may be activated.

The identified differential proteins of hUC-MSCs cryopreserved and thawed at P4 and P10 were enriched in the biological processes pathways of GO classification including differentiation, immunoregulation, wound healing and regeneration, apoptotic signaling pathway, oxidation resistance, cartilage development, regulation of cytokine production, cell migration, aging and others as shown in Table 1, and some proteins were enriched and appeared multiple times in various signaling pathways of hUC-MSCs biological processes including STC1 (Stanniocalcin-1), TNFAIP3 (Tumor necrosis factor alpha-induced protein 3), SERPINE1, COL1A1 (Collagen alpha-1(I)), PDGFR (Platelet-derived growth factor receptor), NCAM1 (Neural cell adhesion molecule 1), C3, JUN (Transcription factor AP-1), GATA6 (Transcription factor GATA-6), HGF (Hepatocyte growth factor), F3 and other proteins likely be used as markers to evaluate hUC-MSCs after cryopreserving and long-term culturing. MSCs can secrete STC1 to protect cancer cells from apoptosis by reducing reactive oxygen radical (ROS), it suggests that STC1 play an important role in antioxidant activity of MSCs [47]. The deficiency of TNFAIP3 in MSCs can induce immune thrombocytopenia and influence megakaryocytic differentiation through terminating the NF-κB pathway that suggests TNFAIP3 play a critical role in the process of MSCs alleviate s autoimmune disease [48]. The mutation of COL1A1 and COL1A2 in MSCs could cause osteogenesis imperfecta, it likely that COL1A1 and COL1A2 play an important role in osteogenesis differentiation from MSCs [49]. PDGFR signaling is emerging as a critical regulatory mechanism and important therapeutic target that critically directs the fate of mesenchymal stem cells during postnatal neovascularization [50]. It is reported that JUN not only can regulate human bone marrow MSCs differentiates into neuron-like cells and acilitates neurite outgrowth, but also play a key role in human MSCs aging and therapeutic potency maintaining [51, 52]. C3 was secreted from MSCs that has an important role in the immunomodulatory and liver regeneration [53, 54]. HGF may have an important role in MSC recruitment sites of tissue regeneration, and may be beneficial in tissue engineering and cell therapy employing hMSCs [55]. These proteins such as STC1, TNFAIP3, SERPINE1, COL1A1, PDGFR, C3, JUN and HGF present important roles in maintaining MSCs function, and CHEK1, SERPINE1, PDGFRB and JUN were also enriched in aging pathway of MSCs biological process. Therefore, these proteins may be used as indicators for the detection of MSCs after cryopreservation and long-term culturing. However, whether these proteins can be used as markers in clinical detection remains to be further studied.

## Conclusion

The morphology, surface markers and tri-lineage differentiation potential of P4 and P10 hUC-MSCs were tested after cryopreservation and a sub-culturing for 24 h and 48 h which was compared with non-cryopreservation and sub-culturing 24 h and 48 h, and the results showed no obvious differences among these groups. However, the proteomics analysis found that cryopreservation leads to changes in a large number of proteins expression compared to those of the controls. This report is the first to show the different effects of freeze-thaw and long-term culture on the proteome of hUC-MSCs. These results will be beneficial to understand the biological process involved in the cryopreservation and long-term culture of hUC-MSCs and contribute to improved cryopreservation protocols that maintain proteomic identity for clinical research, and promote scientists’ attention to the recovery of main proteins and MSCs function after cryopreservation. This will also provide a foundation for safety detection and standardization guide of hUC-MSCs applications in clinical.

## Supplementary information

**Additional file 1: Table S1.** The raw data of pathways and differentially expressed proteins enriched in KEGG database among the eight groups.

## Data Availability

All data generated or analyzed during this study are included in this article.

## Abbreviations

hUC-MSCs: Human umbilical cord-derived mesenchymal stem cells
KEGG: Kyoto Encyclopedia of Genes and Genomes
DMSO: Dimethyl sulfoxide
GO: Gene Ontology
iTRAQ: Isobaric tags for relative and absolute quantification
TMT: Tandem mass tags
PRM: Parallel reaction monitoring
P4N24: Non-cryopreserved and sub-cultured for 24 h at P4
P4C24: Cryopreserved and sub-cultured for 24 h at P4
P4N48: Non-cryopreserved and sub-cultured for 48 h at P4
P4C48: Cryopreserved and sub-cultured for 48 h at P4
P10N24: Non-cryopreserved and sub-cultured for 24 h at P10
P10C24: Cryopreserved and sub-cultured for 24 h at P10
P10N48: Non-cryopreserved and sub-cultured for 48 h at P10
P10C48: Cryopreserved and sub-cultured for 48 h at P10
